# Planetary health: Real challenges for elderly population

**DOI:** 10.1016/j.clinsp.2026.100911

**Published:** 2026-03-23

**Authors:** Fulvio A. Scorza, Josef Finsterer, Antonio-Carlos G. de Almeida, Marcelo F. de Mello

**Affiliations:** a Instituto Israelita de Ensino e Pesquisa, Faculdade Israelita de Ciências da Saúde Albert Einstein, Hospital Israelita Albert Einstein, São Paulo, SP, Brazil; b Disciplina de Neurociência, Departamento de Neurologia e Neurocirurgia, Escola Paulista de Medicina/Universidade Federal de São Paulo (EPM/UNIFESP), São Paulo, SP, Brazil; c Department of Neurology, Neurology and Neurophysiology Center (NNC), Vienna, Austria; d Laboratório de Neurociência Experimental e Computacional, Departamento de Engenharia de Biossistemas, Universidade Federal de São João del-Rei (UFSJ), São João del-Rei, MG, Brazil; e Departamento de Psiquiatria, Escola Paulista de Medicina/Universidade Federal de São Paulo (EPM/UNIFESP), São Paulo, SP, Brazil

Planetary health, first described in 2015, is an interdisciplinary approach that examines the impacts of human-caused disruptions on the environment and the resulting consequences for human health.^1^^,^^2^ In light of this new reality, climate change and extreme weather events, air and water pollution, and toxic chemicals represent environmental problems with catastrophic consequences for human health.^1^ Although these environmental changes affect people of all ages, older adults are particularly vulnerable, and it is crucial to address the specific challenges they face.^3^, ^4^, ^5^, ^6^ Indeed, the world’s population is ageing, a global trend.^7^ Declining birth rates and increasing life expectancy, are leading to a rapid aging of the populations worldwide.^4^ The number of people over 65-years worldwide, which stood at 703 million in 2019, is projected to double to 1.5 billion by 2050.^8^ As the population ages, the physiological and physical functions of some older adults’ decline, making them more vulnerable to environmental impacts.^3^^,^^9^

Climate change is among the greatest global health threats in the 21^st^ century.^6^^,^^10^ The United Nations Framework Convention on Climate Change (UNFCCC) defines climate change as “a change in climate that is directly or indirectly attributable to human activities that alters the composition of the global atmosphere and that is in addition to natural climate variability observed over comparable time periods”.^6^^,^^10^^,^^11^ Therefore, this environmental crisis has significant implications on older adults for several reasons.

First, age-related chronic conditions (e.g., cardiovascular disease, hypertension, obesity, type 2 diabetes, chronic kidney disease, dementia) increase vulnerability to climate change in many older adults.^4^^,^^12^ For example, it is well established that extreme weather conditions (e.g., heat and cold waves) can cause widespread injury, adverse health effects, and death.^13^, ^14^, ^15^ In older people, a heat wave leads to increased blood flow, heat loss, and water loss, ultimately resulting in increased blood viscosity and electrolyte imbalance. Conversely, a cold wave increases the excitability of the sympathetic nervous system, leading to an increase in heart rate, blood pressure, and heart failure.^15^, ^16^, ^17^

Secondly, older people are particularly vulnerable to the effects of Air Pollution (AP).^15^^,^^18^ AP is a major public health problem worldwide, causing over 4 million deaths annually from outdoor pollutants, 2.3 million from indoor air pollution, and a host of cardiovascular, respiratory, and neurological diseases.^18^, ^19^, ^20^ Among older adults, the greatest health impacts and increased mortality occur in people with pre-existing chronic diseases such as asthma, chronic obstructive pulmonary disease and heart disease.^18^, ^19^, ^20^

Third, ticks and mosquitoes will expand their range due to climate change and rising temperatures, population growth, urbanization, international travel and trade, a lack of vector control infrastructure/services and less developed health systems.^4^^,^^15^^,^^21^^,^^22^ Therefore, the likelihood of being bitten by disease-carrying ticks and mosquitoes is increasing.^4^ In this context, some diseases such dengue, zika and Chikungunya viruses are particularly dangerous for older people in Brazil.^4^^,^^15^^,^^23^

Fourth, extreme weather events resulting from climate change (floods, droughts, cyclones and typhoons) have been proven to damage the environment in many ways and certainly increase mortality and disability rates.^15^^,^^24^^,^^25^ Natural disasters have a drastic impact on the physical and mental health of the older people (e.g. post-traumatic stress disorder), as well as on their socioeconomic status.^15^^,^^24^^,^^25^

Fifth, it is important to talk about water crisis and diseases caused by contaminated water. Water is essential for life as it is the main component of cells, tissues and organs and makes up 50%–70% of a young adult’s body weight.^26^^,^^27^ Adults with sedentary lifestyle are advised to drink an average of 2l of water daily.^26^^,^^27^ Despite its undeniable importance, adequate water intake is often overlooked in dietary recommendations (especially in the elderly), leading to dehydration.^26^^,^^27^ In fact, dehydration is one of the leading causes of hospital admission for adults aged 65–75 years.^26^^,^^28^ Despite these important physiological aspects, water pollution is increasing worldwide every year.^29^ The most significant water contaminants that affect the human body are microorganisms and chemicals (pesticides, Polychlorinated Biphenyls [PCBs], phthalates, and Bisphenol A [BPA]). Unfortunately, several important studies have shown that these water contaminants are associated with various health changes in older adults, including cardiovascular, neurological, psychiatric, skeletal and endocrine problems.^29^ Worldwide, agriculture has become increasingly dependent on the synthetic chemical industry in recent decades, which produces patented fertilizers, pesticides, and seeds.^30^ In this sense, humans and their environment are heavily contaminated by synthetic chemical substances used in agriculture.^30^ Disastrously, numerous adverse health effects are associated with exposure to chemical pesticides, such as dermatological, respiratory, reproductive, endocrine, obstetric (child growth), carcinogenic, gastrointestinal and neuropsychiatric consequences.^30^ The situation of older people, is also worrying. Several studies have clearly shown that long-term exposure to pesticides is associated with cognitive decline, the development of neurodegenerative diseases and neuropsychological impairment.^31^ Importantly, recent studies on the climate crisis on mental health provide evidence of increased stress and anxiety, depressive symptoms, cognitive deficits, and sleep disturbances in older people.^32^^,^^33^

Overall, our research group believes that the environmental crisis is increasingly impacting the health of older people worldwide. Therefore, we agree that immediate action is needed, including improvements to early warning systems, health infrastructure, urban planning, social support, and civic engagement.^34^ Finally, the recent consensus on climate and health establishes that transforming resolutions into results requires a close and powerful convergence between governments, international institutions and the Academies of Medicine and medical divisions of Academies of Sciences.^35^ Healthy aging is a modern trend these days and simply wonderful.

## Declaration of competing interest

The authors declare no conflicts of interest.
